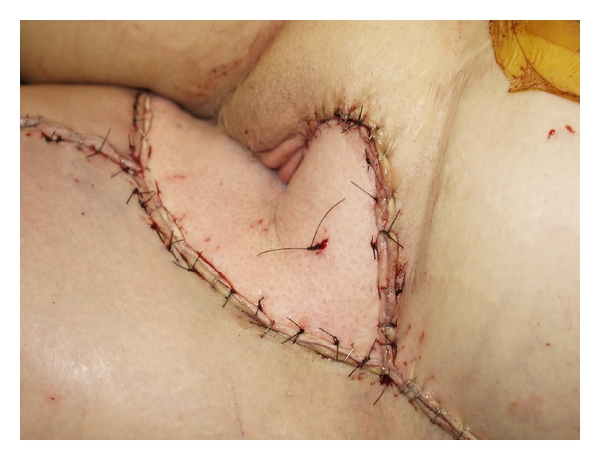# Pedicle Anterolateral Thigh Flap Reconstruction after Pelvic Tumor Resection: A Case Report

**DOI:** 10.1155/2010/684806

**Published:** 2010-11-11

**Authors:** Robert M. Whitfield, David King, Peter Rossi, Michael Loffredo

**Affiliations:** ^1^Department of Plastic Surgery, Medical College of Wisconsin, 8700 Watertown Plank Road, Milwaukee, WI, 53226, USA; ^2^Department of Orthopaedic Surgery, Medical College of Wisconsin, Milwaukee, WI 53226, USA; ^3^Department of Surgery, Medical College of Wisconsin, Milwaukee, WI 53226, USA

## Abstract

A 47-year-old female with a locally advanced urologic malignancy previously managed with resection, diversion, and postoperative radiation therapy presented for management of her recurrent cancer that had eroded through the soft tissues of the left inner thigh and vulva. On all staging studies the tumor involved the left common femoral artery, and vein, both above and below the inguinal ligament. The difficulty with such tumors is the availability of tissue to reconstruct the defect. The patient had a history of deep venous thrombosis in the femoral venous system. A local flap was the most logical type of reconstruction. The patient had a right lower quadrant ureterostomy with a large parastomal hernia which further limited the local flap options. An anterolateral thigh flap from the opposite thigh was used to reconstruct the soft tissue deficit in this patient. This resurfaced the defect and provided coverage for the vascular reconstruction.

## 1. Background

Options are limited for soft tissue reconstruction after tumor removal in the inguinal region. Once the lower extremity has been made ischemic for tumor removal the options for local flap reconstruction are limited. Here a case is presented for the use of a contralateral local flap after tumor removal and prosthetic vascular reconstruction.

## 2. Case Presentation

A 47-year-old female presented with a large foul smelling wound of the left inguinal region with severe pain with ambulation. She had been treated with radiation therapy for a locally aggressive urologic malignancy with not distant metastasis. A surgical plan that involved resection of the tumor, femoral artery, and vein with immediate reconstruction of the vessels and soft tissues was designed for this patient. The abdomen would not be a donor site given her ureterostomy on the right and tumor involvement of both the femoral arterial and venous bifurcations on the left.

The patient underwent resection of the tumor and immediate reconstruction of the femoral artery and vein with heparin-bonded PTFE grafts (Propaten, W.L. Gore, Inc., Flagstaff, AZ) [1]. Once flow had been established to the left lower extremity, the resulting soft tissue deficit was evaluated. An 8 cm × 15 cm soft tissue defect in the inguinal region had to be reconstructed. Given the fact that the patient had a history of deep venous thrombosis, resection of the gracilis with the tumor, and reconstruction of the femoral artery and vein from just below the iliac bifurcations to the midthigh we chose to use the anterolateral thigh flap from the opposite extremity. Other flaps such as the rectus abdominis and rectus femoris are other viable options. In this particular case the diep inferior epigastric vessels were part of the resection. The rectus femoris flap is also useful in reconstruction of groin defects. It can be used without the loss of knee extension. The anterolateral thigh is extremely versatile with multiple uses for lower extremity reconstruction [2, 3]. 

**Figure 1:**
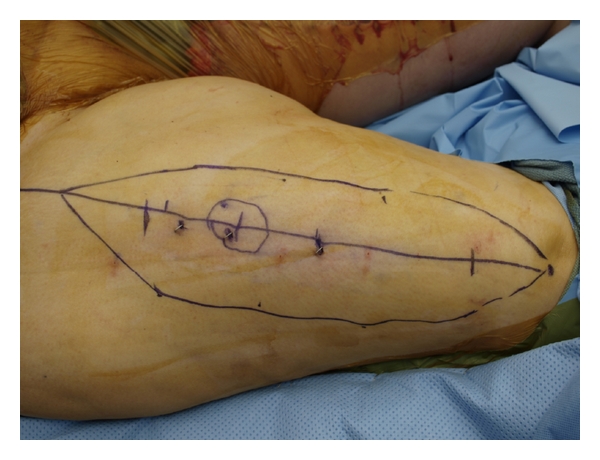


**Figure 2:**
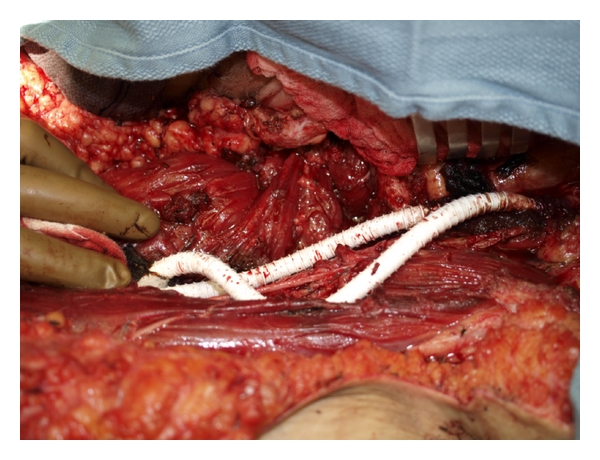


The flap was designed on the lateral thigh in order to obtain maximal pedicle length (Figure 1). Skin signals were identified and marked. An exploration incision was made to identify the descending branch off the lateral circumflex femoral artery. The most distal perforators were located and marked on the surface of the skin paddle of the flap. Typical pedicle length for this flap is approximately 12 cm, depending on the location of the flap [4]. The perforator was the dissected retrograde through the vastus lateralis to the source vessels. The pedicle was dissected to the rectus femoris perforator cranially. Once the dissection was completed, the flap was passed beneath the rectus femoris muscle prior to passage through a subcutaneous tunnel to the defect (Figure 2). Approximately 17 cm of pedicle length was created to facilitate the transfer. The flap was inset and wounds closed over closed suction drains (Figure 3). The patient has gone on to heal her wounds. She is ambulatory and pain free with no evidence of local recurrence. Her venous graft has occluded, but her limb swelling is unchanged from her preoperative status. Her arterial reconstruction remains patent.

## 3. Conclusions

The anterolateral thigh flap has been described as a versatile, thin, pliable, free flap [5]. Given the long pedicle length it is ideal for reaching the ipsilateral groin, pelvis, and contralateral groin provided that adequate pedicle length can be developed prior to the perforating vessels to the rectus femoris muscle. A disadvantage of this flap is the need for skin grafting of the donor site when larger skin paddles are designed. In this case 17 cm of pedicle length was obtained prior to tunneling the flap. With this extralength of approximately 5 cm, the flap was easily inset into the inner thigh of the opposite thigh. For this complicated reconstruction the anterolateral thigh flap proved invaluable [6]. When free flap reconstruction is not ideal such as in cases of metastatic cancer, previous or current deep venous thrombosis, or cases of severe, acute infections, the anterolateral flap as a pedicle rotational flap is a safe and valuable option.

## Figures and Tables

**Figure 3:**